# Reverse total shoulder arthroplasty for treatment of ochronotic arthropathy

**DOI:** 10.1093/jscr/rjac190

**Published:** 2022-04-27

**Authors:** Brian P Butterfield, Thomas H Garver

**Affiliations:** Medical College of Wisconsin, Wausau, WI, USA; Department of Orthopedic Surgery, Marshfield Medical Center, Weston, WI, USA

## Abstract

Ochronotic arthropathy is a chronic manifestation of disease in patients with alkaptonuria. Connective tissue and large joint spaces accumulate discolored homogentisic acid aggregates, resulting in joint degeneration of the axial and appendicular skeleton. One case of right shoulder ochronotic osteoarthritis treated with joint replacement is presented. To our knowledge, this is the first reported surgical intervention of ochronotic arthropathy using the reverse total shoulder arthroplasty technique.

## INTRODUCTION

Alkaptonuria is a rare, autosomal recessive disorder of phenylalanine and tyrosine metabolism. Mutation of the gene encoding homogentisate 1,2-dioxygenase (HGD) causes loss of enzymatic functionality and consequent buildup of homogentisic acid (HGA) [1]. Disease manifestation does not occur until 99% loss of functional HGD activity, with delayed onset of symptoms classically presenting after several decades of disease progression [2].

In the natural course of disease, patients develop alkaptonuric ochronosis. This condition is characterized by polymerization of HGA into brown-black (‘ocher-like’) pigments, causing tissue discoloration. Clinically, ochronosis is often observed in the pinnae of ears, sclera, cornea and conjunctiva. Occasional discovery of ochronosis occurs in patients undergoing joint replacement surgery through direct visualization of discolored synovium and cartilage [3].

Ochronotic arthropathy is an advanced disease state in patients with alkaptonuria. Connective tissue is particularly targeted by chronic accumulation of HGA. Ochronotic plaques within collagen-rich cartilage cause joint rigidity and degeneration through mechanisms not fully understood [2, 4]. The highest incidence of symptoms occurs in the fifth decade of life, with spinal arthropathy and subsequent degeneration of the knee, hip and shoulder joints [2]. This article presents a case of reverse total shoulder arthroplasty in a patient with severe ochronotic osteoarthritis.

## CASE REPORT

A 54-year-old male with medical history of alkaptonuria and ochronosis presented to orthopedics for evaluation of chronic right shoulder pain and stiffness. The patient reported deteriorating right shoulder mobility and arthralgia for the past 5 years. He had no known precipitating physical injuries or overuse events. The patient’s employment did not require physically strenuous activities. His symptoms were refractive to conservative treatment with ibuprofen. Family history was significant for two brothers with alkaptonuria and ochronosis. Both brothers underwent surgical intervention for definitive treatment of ochronotic arthropathy. The first brother was status posttotal knee and total shoulder arthroplasty; the second brother was status postreverse total shoulder arthroplasty.

Physical examination of the patient’s right extremity found significant weakness with applied stress against the supraspinatus, subscapularis and infraspinatus. Stress against the deltoid elicited a normal muscular contour with excellent strength. The acromioclavicular joint was nontender. Patient could slowly elevate his right extremity above his head with assistance. Crepitus was present with active and passive motion in the shoulder.

**Figure 1:**
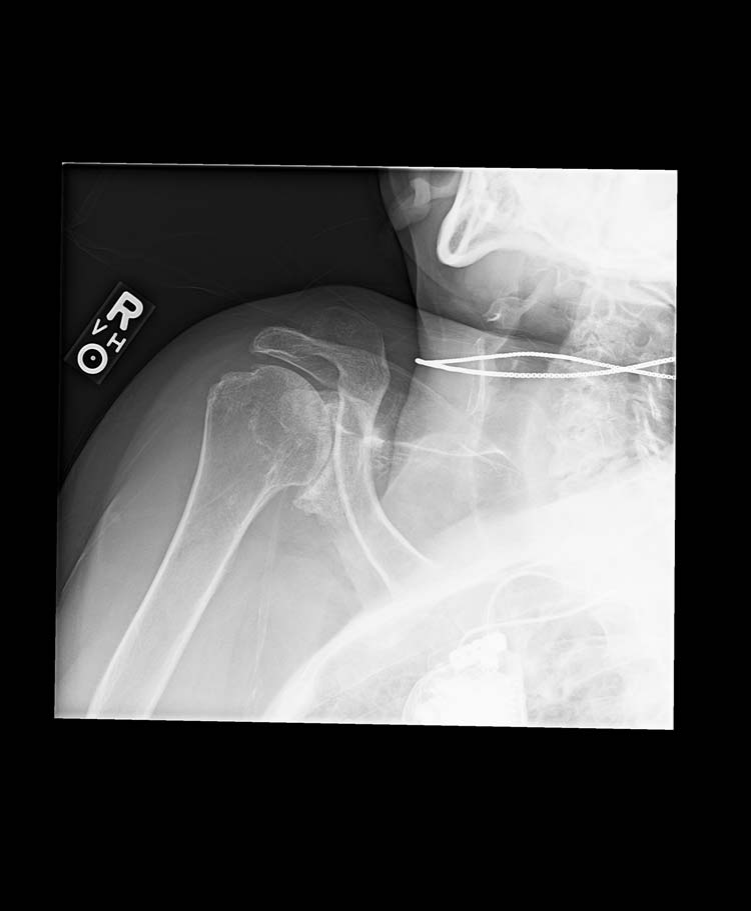
Grashey view demonstrating glenohumeral osteoarthritis with acromiohumeral space narrowing and superior humeral head migration.

**Figure 2:**
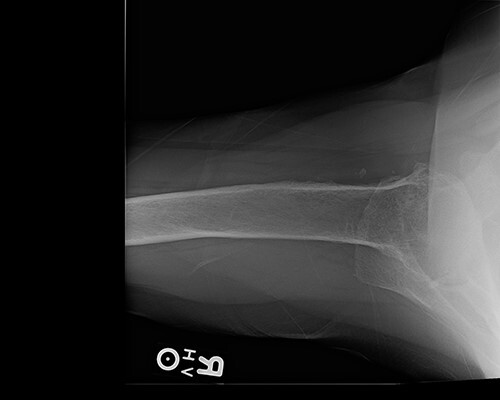
Axillary view demonstrating glenohumeral osteoarthritis.

Radiologic imaging revealed bone-on-bone apposition of the glenohumeral joint, with superior humeral head migration consistent with rotator cuff tear arthropathy (Figs 1 and 2).

The patient was diagnosed with right shoulder rotator cuff tear arthropathy and scheduled for reverse total shoulder arthroplasty.

**Figure 3:**
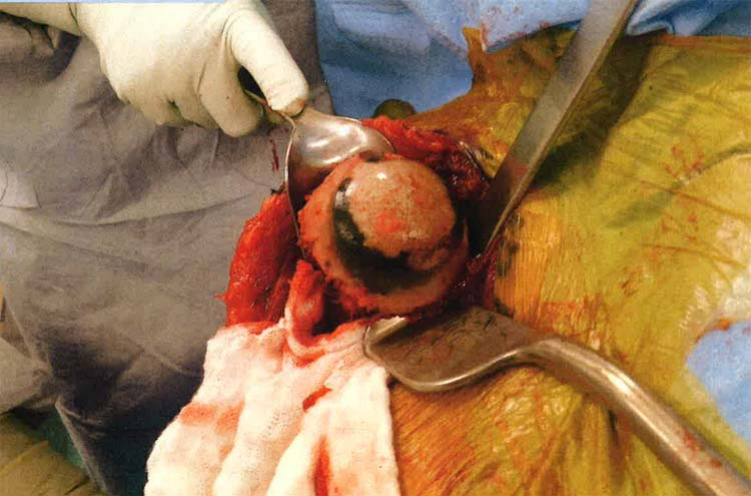
Annular ochronotic pigmentation of right humeral head articular surface.

Operative findings revealed an essentially absent subscapularis, supraspinatus, and infraspinatus. The tendon of long head of bicep was hypertrophied and flattened, trying to act as the rotator cuff. Tenotomy was performed at the level of the superior aspect of the pectoralis insertion on the humeral shaft. The humeral head articular surface was hardened with notable black pigmentation around the periphery (Fig. 3). The glenoid articular surface was essentially denuded of articular surface cartilage.

Surgically implanted devices included the DePuy France Delta Xtend Total Shoulder System, standard metaglene, standard glenosphere, cementless modular humeral stem, cementless modular epiphysis, standard humeral polyethylene cup, locking metaglene screw and non-locking metaglene screw.

**Figure 4:**
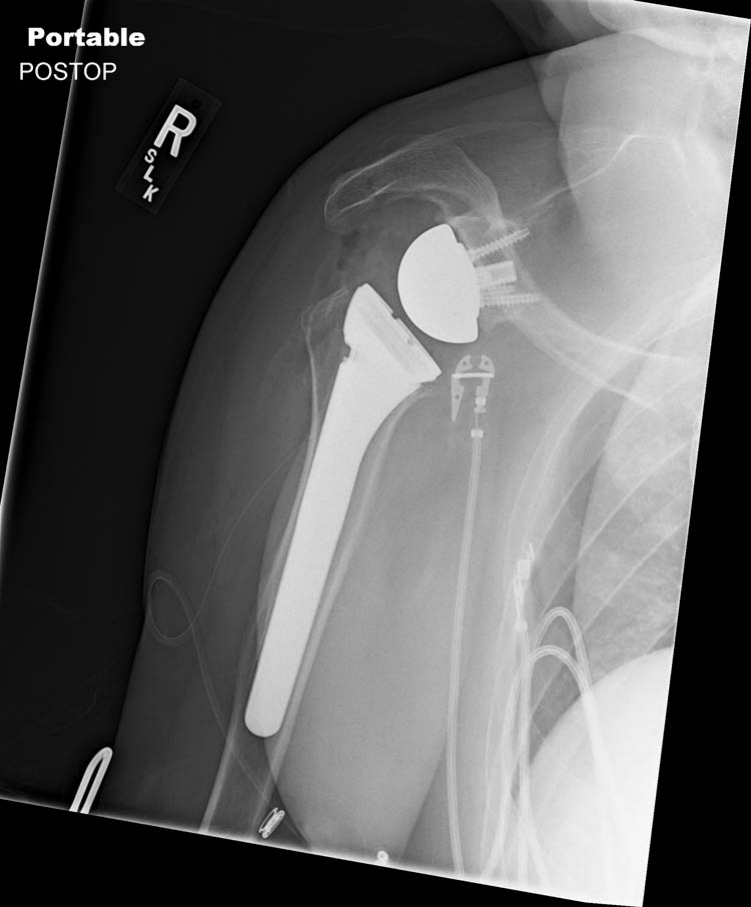
Postoperative Grashey view.

**Figure 5:**
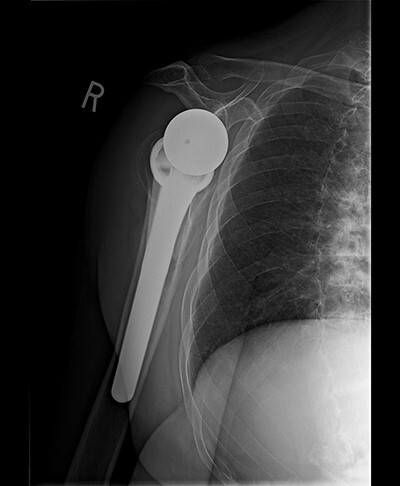
Postoperative Scapular-Y view.

Standard metaglene was positioned superiorly within the glenoid fossa to accommodate a 145° epiphysis and +4-mm lateralized glenosphere (Figs 4 and 5). There were no intraoperative complications.

At the 4-month postoperative follow-up, the patient reported a maximum pain level of 0.5 out of 10. Physical examination demonstrated an active forward elevation of 160° and an active abduction of 120°.

## DISCUSSION

There is no curative treatment for alkaptonuria. Therapy is focused on symptom management and limiting disease progression. Protein restrictive dietary modifications, antioxidant vitamin supplementation and physiotherapy have indeterminate results and unproven efficacy [5]. Joint replacement surgery remains the decisive intervention for degenerative chronic manifestations of this disease.

Reverse total shoulder arthroplasty was approved in 2004 as a definitive treatment modality for osteoarthritis and rotator cuff tear arthropathy. In reverse prosthesis of the shoulder joint, the humerus is fitted with a humeral stem ‘socket’ that pivots around a metal glenosphere ‘ball’ fixed within the glenoid fossa. In contrast to anatomical total shoulder arthroplasty, reverse total shoulder arthroplasty does not require an intact rotator cuff for successful restoration of functional range of motion [6]. While surgical applications for reverse total shoulder prothesis have expanded, there is limited information regarding clinical outcomes in the context of patients with ochronotic osteoarthritis secondary to alkaptonuria.

Findings from this case report suggest that reverse total shoulder arthroplasty is an effective procedure for alleviation of joint pain and mobility improvement in patients with ochronotic arthropathy.

## CONFLICT OF INTEREST STATEMENT

None declared.
